# Hospital-Level Implementation Barriers, Facilitators, and Willingness to Use a New Regional Disaster Teleconsultation System: Cross-Sectional Survey Study

**DOI:** 10.2196/44164

**Published:** 2023-06-27

**Authors:** Tehnaz Boyle, Krislyn Boggs, Jingya Gao, Maureen McMahon, Rachel Bedenbaugh, Lauren Schmidt, Kori Sauser Zachrison, Eric Goralnick, Paul Biddinger, Carlos A Camargo Jr

**Affiliations:** 1 Department of Pediatrics Boston Medical Center Boston, MA United States; 2 Department of Emergency Medicine Massachusetts General Hospital Boston, MA United States; 3 Department of Emergency Management Boston Medical Center Boston, MA United States; 4 Center for Disaster Medicine Massachusetts General Hospital Boston, MA United States; 5 Department of Emergency Medicine Brigham and Women's Hospital Boston, MA United States

**Keywords:** disaster medicine, disaster, telemedicine, telehealth, eHealth, teleconsultation, remote consultation, health care delivery, e-consult, notification, alert, emergency, health system, hospital management

## Abstract

**Background:**

The Region 1 Disaster Health Response System project is developing new telehealth capabilities to provide rapid, temporary access to clinical experts across US jurisdictions to support regional disaster health response.

**Objective:**

To guide future implementation, we identified hospital-level barriers, facilitators, and willingness to use a novel regional peer-to-peer disaster teleconsultation system for disaster health response.

**Methods:**

We used the National Emergency Department Inventory-USA database to identify all 189 hospital-based and freestanding emergency departments (EDs) in New England states. We digitally or telephonically surveyed emergency managers regarding notification systems used for large-scale no-notice emergency events, access to consultants in 6 disaster-relevant specialties, disaster credentialing requirements before system use, reliability and redundancy of internet or cellular service, and willingness to use a disaster teleconsultation system. We examined state-wise hospital and ED disaster response capability.

**Results:**

Overall, 164 (87%) hospitals and EDs responded—126 (77%) completed telephone surveys. Most (n=148, 90%) receive emergency notifications from state-based systems. Forty (24%) hospitals and EDs lacked access to burn specialists; toxicologists, 30 (18%); radiation specialists, 25 (15%); and trauma specialists, 20 (12%). Among critical access hospitals (CAHs) or EDs with <10,000 annual visits (n=36), 92% received routine nondisaster telehealth services but lacked toxicologist (25%), burn (22%), and radiation (17%) specialist access. Most hospitals and EDs (n=115, 70%) require disaster credentialing of teleconsultants before system use. Among 113 hospitals and EDs with written disaster credentialing procedures, 28% expected completing disaster credentialing within 24 hours, and 55% within 25-72 hours, which varied by state. Most (n=154, 94%) reported adequate internet or cellular service for video-streaming; 81% maintained cellular service despite internet disruption. Fewer rural hospitals and EDs reported reliable internet or cellular service (19/22, 86% vs 135/142, 95%) and ability to maintain cellular service with internet disruption (11/19, 58% vs 113/135, 84%) than urban hospitals and EDs. Overall, 133 (81%) were somewhat or very likely to use a regional disaster teleconsultation system. Large-volume EDs (annual visits ≥40,000) were less likely to use the service than smaller ones; all CAHs and nearly all rural hospitals or freestanding EDs were likely to use disaster consultation services. Among hospitals and EDs somewhat or very unlikely to use the system (n=26), sufficient consultant access (69%) and reluctance to use new technology or systems (27%) were common barriers. Potential delays (19%), liability (19%), privacy (15%), and hospital information system security restrictions (15%) were infrequent concerns.

**Conclusions:**

Most New England hospitals and EDs have access to state emergency notification systems, telecommunication infrastructure, and willingness to use a new regional disaster teleconsultation system. System developers should focus on ways to improve telecommunication redundancy in rural areas and use low-bandwidth technology to maintain service availability to CAHs and rural hospitals and EDs. Policies and procedures to accelerate and standardize disaster credentialing are needed for implementation across jurisdictions.

## Introduction

### Background

Recent large-scale, no-notice disasters such as mass shootings and tornadoes, and public health emergencies like the COVID-19 pandemic, have exposed how even the most prepared health care systems can be challenged to function effectively in a crisis [1]. To address this problem, the Administration for Strategic Preparedness and Response in the US Department of Health and Human Services launched a new program to develop regional disaster health response systems (RDHRS) as part of the national strategic response plan to support catastrophic disaster medical care [2]. The goal of an RDHRS is to create partnerships among hospitals and health care facilities that support regional (ie, multistate) health care delivery when existing local and state capacity and capability are exceeded by catastrophic events. RDHRS efforts include novel adaptations of telehealth tools and systems to rapidly expand access to highly specialized, disaster-relevant clinical subspecialists on a regional or national level to support medical response in the immediate aftermath of unusual hazards or catastrophic events.

### Importance

Regionalization of routine specialty care, like trauma and pediatric emergency care, has concentrated the available expert workforce in urban settings and academic medical centers [3], exacerbating access disparities in underserved areas [4-6]. Telehealth services have been used to bridge access to specialty care in underserved, often rural, communities, and in low-resource settings [7-10]. Despite technological advancements, legal, regulatory, and administrative barriers and variability in access to reliable high-speed telecommunications infrastructure have continued to limit the development and use of regional telehealth care delivery models in the United States [11-13]. Variability in medical licensing and credentialing procedures has also hindered the ability to leverage clinical expertise across state lines and jurisdictions [12]. Telehealth services have demonstrated use in military disaster settings [14]. However, civilian telehealth systems to support trauma, burn, pediatric, and other highly regionalized specialty care remain generally underdeveloped and inaccessible to many community hospitals that may be overwhelmed in disaster events [15,16].

### Goals of This Investigation

The Region 1 RDHRS, which covers 6 New England states, is developing a new peer-to-peer disaster teleconsultation system to support disaster health response across state lines [17]. This system is designed to rapidly expand regional access to disaster-relevant medical experts (eg, burn surgeons), who may be in limited supply immediately following a large-scale, no-notice emergency event, such as a mass casualty incident. The goal is not to replace existing telehealth services but to supplement overwhelmed services or provide new ones where they are otherwise unavailable when needed in a crisis. The RDHRS disaster teleconsultation system aims to provide local bedside providers access to electronic consultation services with remote clinical specialists via telephone, video, or other platforms compliant with regulatory standards governing protected health information within hours of an event [18]. This would allow disaster-relevant clinical experts to participate early in the acute response phase before in-person disaster teams can be mobilized and deployed to the field. To guide development and future implementation, the objectives of this study were to identify barriers and facilitators to implementation and to determine willingness (primary outcome) to use a new regional disaster teleconsultation system at a hospital level in New England.

## Methods

### Study Design and Setting

We conducted a cross-sectional survey study of all hospital-based and freestanding emergency departments (EDs; hospitals and EDs) open in March 2021 in the 6 New England states (Connecticut, Maine, Massachusetts, New Hampshire, Rhode Island, and Vermont).

### Ethical Considerations

For this study, we collected only facility-level information about hospitals and EDs. No compensation was provided for the completion of the study. All data are securely stored on a password-protected server accessible to the study staff. The Mass General Brigham Human Research Committee reviewed this project and classified it as exempt.

### Survey Administration

Staff from the Emergency Medicine Network (Boston, Massachusetts) used the National Emergency Department Inventory (NEDI)-USA database to identify all 189 New England hospitals and EDs open during the study period. Briefly, NEDI-USA contains information about all nonfederal, nonspecialty US EDs [19]. Between March 1 and June 30, 2021, we called the main telephone number for each hospital or ED and requested to speak to the emergency manager for survey completion by structured interview. We selected emergency managers as key respondents, as these individuals are responsible for and most knowledgeable about the disaster preparedness and response capabilities of their facilities.

Three staff members with formal training and prior experience with contacting hospitals and EDs for other surveys about facility characteristics conducted the surveys. All used a standardized protocol and interview guide with scripted explanations in case respondents requested clarification of terms and definitions. Telephone survey responses were directly entered into a secure database by the interviewer, using Research Electronic Data Capture (REDCap; Vanderbilt University) tools hosted at Massachusetts General Hospital [20]. REDCap is a secure, web-based software platform designed to support data capture for research studies. We also created a web-based version of the survey administered via REDCap as an alternate means of participation. The web-based survey link was active throughout the entire course of data collection.

Participants were given the option to either complete the interview during the first call, schedule a time for callback, or receive the survey by email. All respondents were able to ask clarifying questions by telephone or via email before completing the survey regardless of the mode of survey administration. Survey respondents also had the option to leave questions unanswered or consult other hospital staff (eg, compliance officers) to confirm answers if needed.

### Measurements

The survey questions were developed by the multidisciplinary region 1 RDHRS Telehealth Working Group, which includes experts in adult and pediatric emergency medicine, disaster medicine, public health, hospital emergency system management, and telehealth implementation. Survey questions were reviewed by a hospital credentialing and compliance officer, 2 directors of emergency preparedness, and 2 emergency management specialists to ensure face validity.

The survey collected information about (1) how each hospital and ED receives notification of large-scale or state-wide no-notice emergency events, (2) the availability of 6 disaster-relevant clinical specialist types (toxicology, radiation or nuclear medicine, trauma, burn, high-consequence infectious disease, and critical care) for consultation in-person, via telephone, or via telehealth within 4 hours of an event, (3) disaster credentialing requirements of remote specialists before system use, (4) availability of internet or cellular service to support video streaming in clinical spaces, and (5) whether each hospital and ED has a contingency plan if internet or cellular service is disrupted. To assess hospital-level willingness (primary outcome), we asked respondents how likely it would be for their hospital and ED to use a new disaster teleconsultation system to access specialists (very likely, somewhat likely, somewhat unlikely, or very unlikely). Among those who were “somewhat unlikely” or “very unlikely” to use this system, we asked about potential barriers to use (Multimedia Appendix 1).

Additionally, using the NEDI-USA data set, we identified each ED’s total annual visit volume (<10,000; 10,000-19,000; 20,000-39,000; ≥40,000), whether or not each ED was a designated critical access hospital (CAH) [21] or freestanding ED [22], whether each hospital and ED was in an urban or rural location based on location within or outside of a core based statistical area, and whether the ED received routine (nondisaster) telehealth services [23].

### Primary Data Analysis

We used descriptive and bivariate statistics to describe and compare the disaster response characteristics of New England hospitals and EDs by state. Data are presented as frequencies with percentages. A chi-square test and the Fisher exact test were used to examine interstate variation in ED disaster response capability. Fisher exact test was used alternatively for low counts. *P* values of <.05 were considered statistically significant. We also created maps to demonstrate the geographic differences in the hospital or ED disaster response infrastructure. Data analysis and mapping were performed using SAS (version 9.4; SAS Institute).

## Results

Of the 189 hospitals and EDs open in New England in 2021, a total of 164 (87%) responded to the survey. Among these, 126 (77%) completed the survey by phone interview, and 38 (23%) completed it digitally. The response rate in each of the 6 New England states was ≥83%. There were no differences in ED characteristics between responders and nonresponders.

Table 1 summarizes the emergency notification mechanisms, whereas Table 2 summarizes gaps in access to disaster-relevant specialists for consultation in New England hospitals and EDs. While the percentage of hospitals and EDs notified by state-level systems or emergency medical services (EMS) and public safety systems was similar across all states, notification by regional- or county-level systems or via media mechanisms was more varied (Table 1). Most New England hospitals and EDs (97%) could access at least 1 type of disaster-relevant specialist for consultation in-person, via telephone, or via telehealth within the first 4 hours of a large-scale, no-notice emergency event (Table 2). There was no significant difference in access to specialists by state.

ED characteristics are summarized in Table 3. Because small volume EDs (<10,000 annual visits), CAHs, and rural hospitals may lack access to specialists, we further examined this subset (Table S1 in Multimedia Appendix 1). Eighteen of the 22 EDs with <10,000 annual visits were also designated CAHs. Among CAHs or EDs with <10,000 annual visits (n=36), 92% received routine nondisaster telehealth services, and most reported access to specialists in trauma (92%), high-consequence infectious disease (92%), and critical care (97%). However, 25% lacked access to toxicologists, 22% to burn specialists, and 17% to radiation or nuclear medicine specialists.

**Figure 1:**
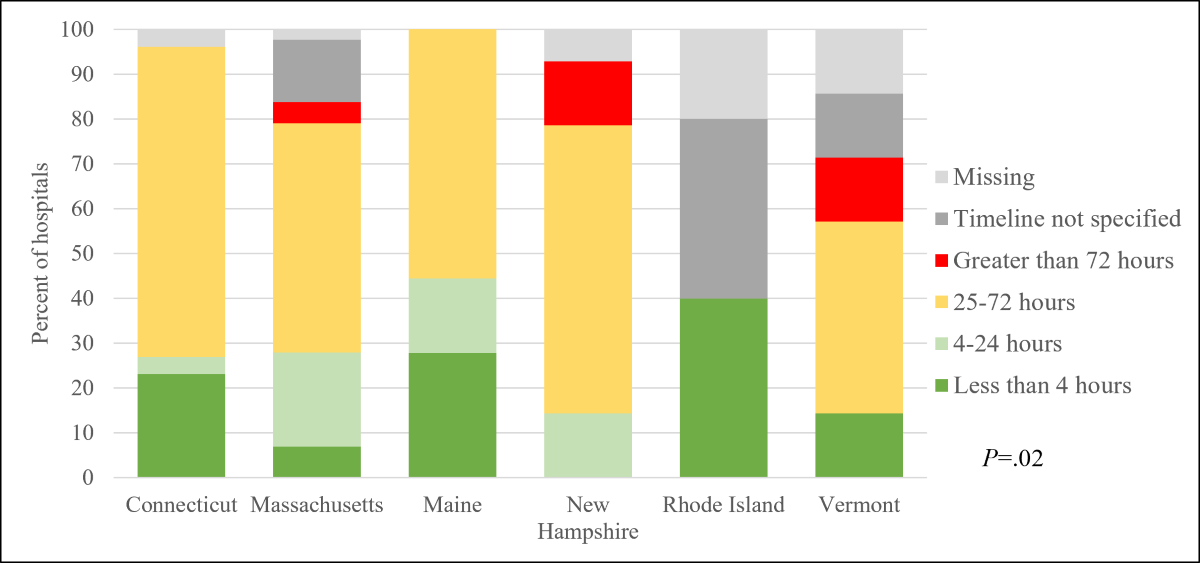
By-state comparison of expected timeline to complete disaster/emergency credentialing and privileging procedures among New England hospitals and emergency departments that would require disaster credentialing and had written procedures in medical staff bylaws (n=113).

Next, we asked if hospitals and EDs would require disaster credentialing of teleconsultants before permitting system use. Overall, 70% (115/164) of New England hospitals and EDs reported that they would require disaster credentialing and 113 of these had written procedures in their medical staff bylaws. Among those with written procedures (n=113), disaster credentialing (n=105, 93%) was used more frequently than credentialing-by-proxy (n=16, 14%) or other procedures (n=5, 4%). Over half (n=62, 55%) expected disaster credentialing to take 25 to 72 hours to complete and varied by state. Only 32 (28%) expected to complete these procedures within 24 hours and 17 (15%) within 4 hours. Figure 1 depicts the variability in expected time to complete disaster credentialing procedures in hospitals and EDs within and between states in New England. Notably, 43 (38%) had used disaster credentialing procedures during a major event in the past 20 years, of which 53% had successfully completed it within 4 hours. Approximately two-thirds of hospitals and EDs requiring disaster credentialing of teleconsultants were willing to use a third-party verification system to complete this procedure. More hospitals and EDs in Rhode Island (100%), Maine (94%), and New Hampshire (86%) were willing to use a third-party verification system than in Vermont (57%), Connecticut (52%), and Massachusetts (47%).

**Figure 2:**
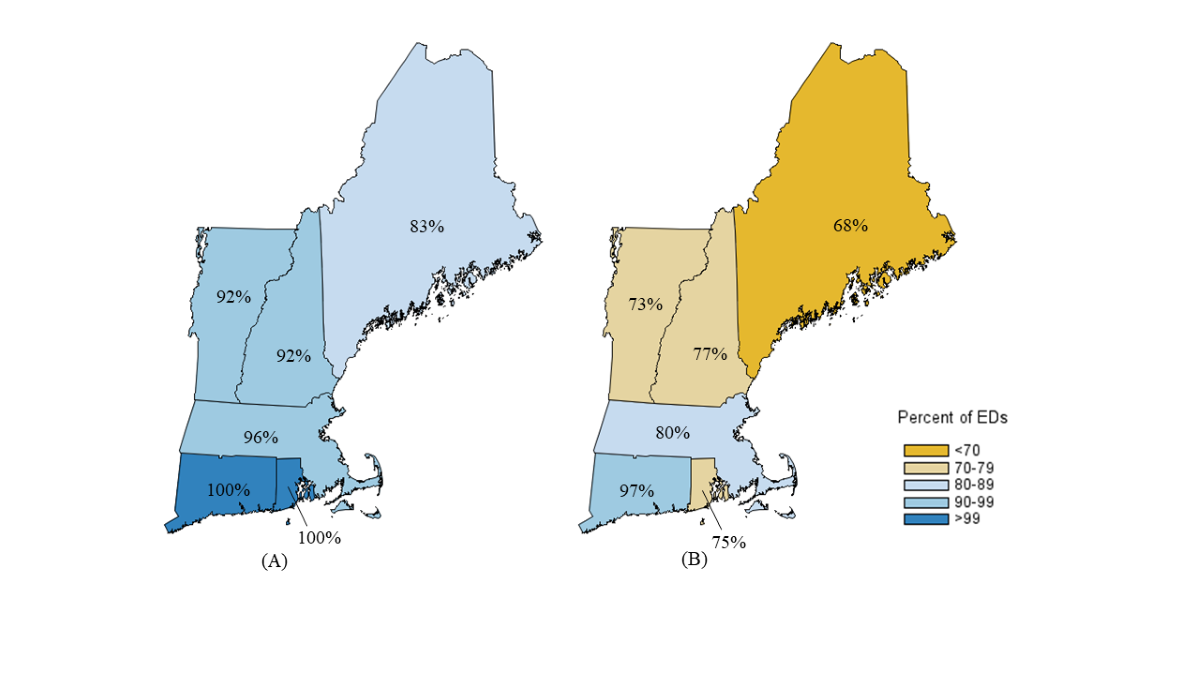
Reliability and redundancy of telecommunications infrastructure. (A) The percentage of emergency departments with adequate internet and cellular service to support video-streaming (reliability). (B) The percentage of emergency departments that maintain cellular service despite internet disruption (redundancy). ED: emergency department.

We then examined the reliability and redundancy of hospital telecommunication infrastructure as this could affect adoption and use of a disaster teleconsultation system. Overall, 154 (94%) hospitals and EDs reported reliable internet or cellular service connectivity for video streaming in clinical areas, and 142 (87%) had a contingency plan for loss of telecommunication services. Of the 154 hospitals and EDs with reliable connectivity, 124 (81%) could maintain cellular service if there was an internet disruption. Hospitals and EDs in Maine, New Hampshire, and Vermont had less reliable telecommunication and lower capability of maintaining services during disruption (Figure 2). Fewer rural hospitals and EDs reported reliable internet or cellular service (19/22, 86% vs 135/142, 95%) and ability to maintain cellular service with internet disruption (11/19, 58% vs 113/135, 84%) than urban hospitals and EDs.

**Figure 3:**
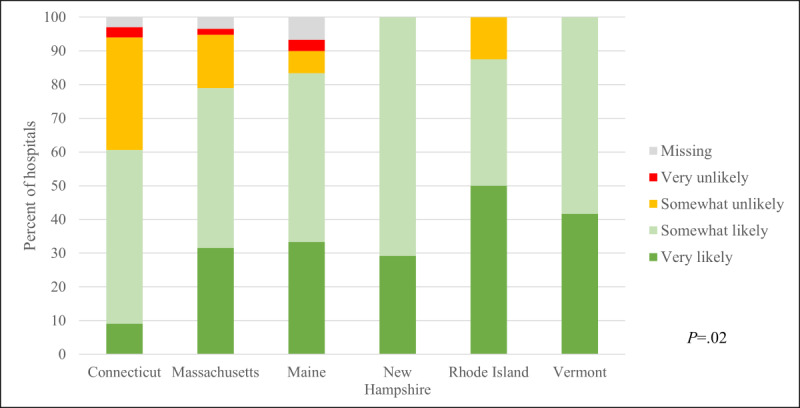
Willingness of New England hospitals and emergency departments to use a regional disaster teleconsultation system to access specialists by state (n=164).

Most (n=133, 81%) New England hospitals and EDs were very or somewhat likely to use RDHRS disaster telehealth services to access specialists if a large-scale no-notice event affected their facility. Hospitals and EDs in Vermont, New Hampshire, and Rhode Island reported higher willingness to use the system than those in Maine, Massachusetts, and Connecticut (Figure 3). Among the 26 (16%) hospitals and EDs that were somewhat or very unlikely to use the system, the leading barriers were sufficient access to specialists in-house or within the health system (n=18, 69%) and reluctance to use new technology or systems during a disaster (n=7, 27%). Potential time delays (n=5, 19%), liability (n=5, 19%), privacy (n=4, 15%), and hospital information system security restrictions (n=4, 15%) were other reported concerns.

Table 3 examines the willingness to use disaster teleconsultation services by ED characteristics. While overall willingness was high, large volume EDs (annual visits ≥40,000) were less likely to use the service than smaller ones. In contrast, all CAHs and nearly all rural hospitals or freestanding EDs were likely to use disaster consultation services. Eighty percent of hospitals and EDs that were likely to use the system also received nondisaster telehealth services.

**Table 1:** Overall and by-state comparison of mechanisms by which New England hospitals and emergency departments are notified of large-scale or state-wide no-notice emergency events.

Disaster response capability	Total (n=164), n (%)	Connecticut (n=33), n (%)	Massachusetts (n=57), n (%)	Maine (n=30), n (%)	New Hampshire (n=24), n (%)	Rhode Island (n=8), n (%)	Vermont (n=12), n (%)	*P* value
EMS^a^ or public safety system notification	142 (87)	32 (97)	47 (82)	27 (90)	21 (88)	7 (88)	8 (67)	.13
Hospital network emergency notification system activated at state level	148 (90)	33 (100)	48 (84)	27 (90)	20 (83)	8 (100)	12 (100)	.10
Hospital network emergency notification system activated at regional or county level within a state	133 (81)	28 (85)	48 (84)	28 (93)	17 (71)	6 (75)	6 (50)	.02
Media notification	80 (49)	29 (88)	24 (42)	12 (40)	8 (33)	2 (25)	5 (42)	<.001
Other^b^	13 (8)	5 (15)	5 (9)	3 (10)	0 (0)	0 (0)	0 (0)	.36

^a^EMS: emergency medical service.

^b^“Other” includes receiving notifications from a dedicated third party, internal notification system, national alert network such as from Centers for Disease Control, and so forth.

**Table 2:** Frequency and percentage of New England hospitals and emergency departments reporting no access to specialists for consultation in-person, via telephone, or via telehealth within first 4 hours of large-scale, no-notice emergency event, overall and by state.

Disaster response capability	Total (n=164), n (%)	Connecticut (n=33), n (%)	Massachusetts (n=57), n (%)	Maine (n=30), n (%)	New Hampshire (n=24), n (%)	Rhode Island (n=8), n (%)	Vermont (n=12), n (%)	*P* value
Toxicology	30 (18)	4 (12)	11 (19)	3 (10)	7 (29)	3 (38)	2 (17)	.27
Radiation or nuclear medicine	25 (15)	3 (9)	8 (14)	7 (23)	4 (17)	2 (25)	1 (8)	.61
Trauma	20 (12)	2 (6)	11 (19)	3 (10)	1 (4)	2 (25)	1 (8)	.19
Burn	40 (24)	3 (9)	20 (35)	8 (27)	5 (21)	2 (25)	2 (17)	.08
Infectious disease	7 (4)	1 (3)	1 (2)	4 (13)	1 (4)	0 (0)	0 (0)	.26
Critical care	6 (4)	1 (3)	2 (4)	3 (10)	0 (0)	0 (0)	0 (0)	.59
No access to any specialist	2 (1)	1 (3)	0 (0)	1 (3)	0 (0)	0 (0)	0 (0)	.52

**Table 3:** Emergency department (ED) characteristics by willingness to use a regional disaster teleconsultation system.^a^

Characteristics			Total (N=164), n (%)		Very likely (N=47), n (%)		Somewhat likely (N=86), n (%)		Somewhat unlikely (N=23), n (%)		Very unlikely (N=3), n (%)
**Annual ED visit volume**											
	<10,000	22 (13)		8 (17)		12 (14)		1 (4)		0 (0)	
	10,000-19,999	46 (28)		8 (17)		32 (37)		4 (17)		1 (33)	
	20,000-39,999	48 (29)		21 (45)		20 (23)		6 (26)		1 (33)	
	≥40,000	48 (29)		10 (21)		22 (26)		12 (52)		1 (33)	
**Critical access hospital**											
	Yes	32 (20)		12 (26)		19 (22)		0 (0)		0 (0)	
	No	132 (80)		35 (74)		67 (78)		23 (100)		3 (100)	
**Freestanding ED**											
	Yes	12 (7)		2 (4)		9 (10)		0 (0)		1 (33)	
	No	152 (93)		45 (96)		77 (90)		23 (100)		2 (67)	
**Urban status**											
	Urban	142 (87)		41 (87)		72 (84)		22 (96)		3 (100)	
	Rural	22 (13)		6 (13)		14 (16)		1 (4)		0 (0)	
**Access to routine (nondisaster) telehealth services**											
	Yes	132 (80)		41 (87)		65 (76)		18 (78)		3 (100)	
	No	17 (10)		2 (4)		11 (13)		4 (17)		0 (0)	
	Missing	15 (9)		4 (9)		10 (12)		1 (4)		0 (0)	

^a^5 participants skipped the willingness question.

## Discussion

### Principal Results

In this regional study, we found that most New England hospitals and EDs had the necessary emergency notification mechanisms, telecommunication infrastructure, and willingness to support the future regional implementation of a disaster teleconsultation system. Although many EDs had access to at least 1 disaster-relevant specialist, 1 in 4 New England hospitals and EDs lacked access to burn specialists, identifying an important gap to target in future service delivery. Rural hospitals, freestanding EDs, and CAHs demonstrated the highest willingness to use this type of service but may require improvements in telecommunication infrastructure to support use in conditions where connectivity is disrupted or infrastructure destroyed. Despite limiting disaster telehealth services to teleconsultation only, 7 in 10 hospitals and EDs would require disaster credentialing before using the service and less than a third of these sites could complete this procedure within 24 hours. This could significantly delay postdisaster access to specialists required for time-critical patient care.

### Comparison With Prior Work

Identifying which emergency notification mechanisms are used most frequently by regional hospitals can help RDHRS programs prioritize the most effective communication channels. RDHRS programs are designed to build on local and state assets and emergency response capabilities and provide regional coordination of key health care resources, such as burn centers and pediatric hospitals. Our findings suggest that integrating RDHRS communication strategies with hospital emergency network notification systems activated by state, EMS, and public safety systems would be the most efficient mechanisms for rapid regional communication. Such channels could be used to notify hospitals of the availability of regional disaster services in the aftermath of an event. Formalizing RDHRS programs as regional response entities within the national strategic response plan would facilitate future integration with existing emergency notification systems at the local and state level.

Depending on regional need and availability, RDHRS programs may need to create new disaster telehealth service lines or facilitate mechanisms to expand access to existing ones. Understanding gaps in regional access to disaster-relevant specialty care is necessary to target which service lines need the most urgent development, especially as rural and urban practitioners may perceive service need and use differently [24]. Among New England hospitals and EDs, we found larger gaps in access to burn, toxicology, radiation, and trauma specialists than to infectious disease and critical care specialists. Burn and trauma care are notable examples where disparities in routine access may be worsened by a disaster. For example, after a mass casualty incident, interfacility patient transfers may be delayed due to limited availability of emergency medical services for transportation, and overwhelming volume may limit capacity at receiving specialty hospitals. Regional disparities in access to burn centers have been described [25,26]. Despite the ability to alter triage, care, and transfer decisions [27-29], many burn telehealth programs remain localized without regional or national coordination due to barriers in sustainable funding and license portability [30,31]. Regional variation in access to trauma centers impacts injury mortality and emergency resource usage [32,33]. While trauma telehealth programs support providers who are uncomfortable or unfamiliar with specialty trauma care and improve transfer efficiency for acutely injured patients [34-36], adoption has lagged.

Mass casualty incidents involving radiological and chemical agents also require immediate access to experts in radiological exposure, hazardous materials, and medical toxicology. Although federal guidance emphasizes early mass decontamination during the emergency response to such incidents [37], a qualitative study across 3 US regions found ED clinicians perceived a lack of hospital preparedness for radiological terrorism [38]. Teleconsultation with hazardous material or radiation specialists could be used to support field providers and receiving hospitals, although studies examining the use of telehealth for such incidents are sparse. Teleconsultation with patients for counseling and risk communication after exposure could add public health benefit, as described after the 2011 Fukushima nuclear disaster [39]. Regional poison centers can also be integrated into disaster response infrastructure to leverage available medical toxicology expertise [40]. Like other service lines with low-frequency use, the adoption of routine teleconsultation with medical toxicologists has been limited by reimbursement barriers [41]. Dedicated funding to ensure the correct people, equipment, and administrative supports are maintained during the preparedness portion of the disaster cycle will be essential to ensure effective national strategic response for future events.

Historically, concerns over the cost of technology and service use, and administrative barriers related to interstate license portability and hospital credentialing and privileging requirements have slowed the expansion of telehealth in the United States [12,42-45]. Licensure refers to the process of securing the authority to practice medicine within a state. Credentialing refers to the administrative process of validating the competency of a health care provider by verifying their license, education, certification, and other information to ensure they meet the practice standards required by a hospital. Disaster credentialing is an expedited procedure that can only be used when a hospital’s emergency operations plan is activated to facilitate rapid access to outside assistance (eg, medical professionals) to sustain patient care. Our findings highlight how variability in regulatory and administrative practices at the state and hospital level could hobble efforts to integrate telehealth tools into regional disaster medical response. Despite limiting disaster services to peer-to-peer teleconsultation with certified expert providers, 70% of New England hospitals and EDs would require disaster credentialing of teleconsultants before permitting service use with many requiring up to 72 hours to complete this process. This administrative barrier could delay implementation by days to weeks and negate the potential benefits of accessing specialists within hours of a no-notice event, like a mass casualty incident, where interventions and outcomes are critically time sensitive.

Our findings are surprising as peer-to-peer teleconsultation for the purpose of providing expert advice is not subject to the same legal and regulatory requirements as telehealth services that deliver direct patient care [11]. Common licensure exceptions include physician-to-physician consultations, public health services, medical emergencies, and natural disasters [11]. For example, Regional Poison Control Centers represent a widely accepted model where episodic, emergency consultations are delivered via telephone by certified expert personnel across jurisdictions [46]. These consultations are freely and routinely accessible to providers without administrative requirements for hospitals using the service. Whether survey respondents perceived expert teleconsultations via a regional disaster health response system as distinct from telephone consultations via a regional poison control center is unclear. Finally, in the absence of clear guidance on acceptable standards, hospital administrators may vary in their approach to disaster credentialing to avoid potential regulatory penalties and minimize medicolegal risk [12]. We encourage future research to understand the reasons underlying the variability in hospital credentialing practices, as this could have important implications for policy makers.

Creation of a national licensure standard, incentivizing the adoption of existing mechanisms to streamline interstate licensure such as the Interstate Medical Licensure Compact [47], or permitting limited exceptions to telehealth licensure laws could all support the ability to maintain a rapidly deployable network of disaster-relevant specialists at the ready. The Centers for Medicare and Medicaid Services could also incentivize hospitals to adopt mechanisms to streamline disaster credentialing procedures by using rapid third-party verification mechanisms. For example, Provider Bridge, a web-based platform to speed verification of out-of-state health care volunteers, was developed by the Federation of State Medical Boards with temporary funding from the Health Resources and Services Administration and the Coronavirus Aid, Relief, and Economic Security Act [48]. However, cessation of grant funding has left the future of such programs uncertain. We strongly support continued funding and intentional integration of effective pilot programs to ensure investments made to support pandemic response are available for future disaster response.

Successful application of telehealth requires reliable access to high-speed broadband networks and functional telecommunications infrastructure. Broadband refers to various technologies (eg, cable, wireless, satellite, wireless, and mobile) that provide high-speed data transmission and connection to the internet. Prior studies have described disparities in access to broadband coverage in the United States, particularly in rural communities and tribal lands [49,50]. By the most recent Federal Communications Commission estimates, 77% of Americans in rural areas and 72% in tribal lands have broadband access via fixed terrestrial (eg, cable) and mobile (cellular) networks at speeds that can support 2-way video-conferencing as compared to 99% of Americans in urban areas [51]. In this study, most New England hospitals and EDs reported adequate broadband access via fixed and mobile networks to support video streaming in clinical spaces. However, 1 in 5 rural hospitals in New England may lose this capability if fixed networks are disrupted because they have inadequate access to mobile networks within facility walls. More research is required to understand regional capabilities to mobilize satellite or mobile towers when infrastructure is lost.

Telehealth platforms that function at low bandwidth speeds and operate on both fixed and mobile networks will be necessary for resiliency in disasters where telecommunications infrastructure may be damaged or destroyed, or networks overwhelmed by surging demand. Additionally, asynchronous communication mechanisms that remain operational in limited bandwidth settings could also be sufficient to serve some disasters and areas. Federal investment in closing the “digital divide” should include strategies to ensure reliability and redundancy of telecommunications infrastructure in vulnerable facilities in rural areas and tribal lands. A similar approach could support telehealth applications for humanitarian response in resource-limited settings and different cultural or geographic environments.

Widespread adoption and use of telehealth during the COVID-19 pandemic has resulted in broad technology acceptance within the global health care community. In the postpandemic environment, cost may become a lesser barrier as clinical needs push providers and organizations to adopt new models of remote care delivery [52]. We found high willingness to use regional disaster teleconsultation services to access key specialist groups among New England hospitals and EDs. This survey was conducted in 2021 after the first year of the pandemic, when many hospitals were still struggling with large patient surges and workforce shortages and using emergency operations [53,54]. While this may have biased respondents toward expressing greater willingness, their attitudes are arguably informed by real-world experience rather than conjecture. Whether frontline providers and clinicians would express similar levels of willingness to use disaster teleconsultation services is unclear, although simulation-based studies suggest this may hold true [17]. Future research examining attitudes and drivers of the adoption of disaster telehealth services on a provider level, mechanisms to disseminate awareness of service availability, and integration with existing emergency management plans will be needed to ensure service lines will actually be used. Finally, even the hospitals and EDs that are willing to use disaster telehealth services may experience barriers to implementation. Thus, system developers should not only indicate potential demotivating factors, barriers, or weaknesses to implementation but also identify critical acceptance criteria to implement disaster telehealth services on a hospital level.

The motivators and challenges to the implementation of regional disaster telehealth systems for cross-jurisdictional response in the United States parallel applications of telehealth for international humanitarian response [55]. The Sendai Framework for Disaster Risk Reduction 2015-2030 was developed to guide international efforts in disaster risk reduction through coordinated implementation of various measures to reduce vulnerabilities, increase preparedness for response and recovery, and strengthen resilience [56]. In 2022, rapidly deployable disaster telehealth services coordinated by an international, nongovernmental collaborative of digital health, telehealth, disaster response, and medical experts and staffed by clinical volunteers across the globe provided access to health care for Ukrainians displaced by the Russia-Ukraine conflict [57]. This was a powerful demonstration of how a disaster telehealth system could leverage a remote international workforce to support large-scale humanitarian response and supplement in-person field response. However, in the United States, many insurers and state licensing boards only permit telemedical practice with oversight due to the risk of potential abuse. Furthermore, lack of adoption of standards or agreements prevents efficient use of telehealth solutions to support timely “surge capacity” to deliver health services during emergencies because expert volunteers are mired in cumbersome and complicated processes of entering into agreements with their home jurisdictions before they can be digitally “deployed” across jurisdictions. We call for the uniform adoption of national (eg, Uniform Emergency Volunteer Health Practitioners Act) and international standards governing licensure and verification of medical volunteers practicing across jurisdictions to facilitate engagement and avoid implementation delays.

### Limitations

This study has potential limitations. First, we defined disaster teleconsultation services as electronic consultations between providers as recognized by the Health Resources and Services Administration [18]. Here, teleconsultants provide peer-to-peer advice only and the bedside clinician makes final patient care decisions. However, some respondents may not have distinguished this from nonconsultative services or other types of routine, nondisaster telehealth services where a remote clinician directs patient care. One possibility is that emergency managers lack knowledge of the credentialing procedures that are the purview of compliance officers and provided erroneous answers. Another possibility is that despite our efforts to define the limited scope of disaster teleconsultation services, respondents were confused by or disagreed with our definitions. If respondents did not distinguish between teleconsultation for advice and telehealth to provide direct patient care, they could perceive credentialing procedures as necessary. Confusion about the service type offered could overestimate variability in disaster credentialing procedures. However, respondents were provided ample opportunity to ask clarifying questions regarding terms and definitions during survey administration, and we did not perceive any confusion to this end.

Second, the data were self-reported by 1 person per site, which may have introduced information bias. However, we mitigated this by contacting emergency managers as respondents, as they are responsible for and knowledgeable about disaster operations and plans for their facilities. Third, the attitudes of hospital emergency managers may not reflect those of frontline clinicians or other hospital administrators, so it is possible our estimates of willingness to use regional disaster telehealth services are inaccurate. Future research should examine the attitudes of additional groups involved in the adoption and implementation of telehealth services for disaster response on a hospital level. Last, this survey was administered in New England, which may not be representative of the other regions. However, diversity in geography and hospital and ED types within the region supports the generalizability of the findings.

### Conclusions

In summary, we found that most hospitals and EDs in New England have the necessary access to statewide emergency notification mechanisms, telecommunications infrastructure, and willingness to use a new regional disaster teleconsultation system to access specialists if a large-scale event affected their facility. Even in a relatively resource-rich region, access to burn care was the most limited among numerous disaster-relevant specialty types and should be an early focus for service line development. We also identified significant variability and lack of standardization in hospital credentialing practices, which could delay timely access to teleconsultants and diminish potential benefits for no-notice disasters with time-sensitive outcomes. These findings can inform how health systems and disaster response organizations can coordinate strategic plans to implement and integrate new telehealth tools for regional disaster medical response in New England. We encourage future research on interregional differences in clinical needs, gaps in telecommunications infrastructure, and reasons for variability in disaster credentialing practices at a hospital level. This research is critical to build an effective telehealth capability that supports real-world needs for regional disaster health care response across the United States.

## Appendix

Multimedia Appendix 1 Survey and supplemental table.

## Abbreviations

CAH: critical access hospital
ED: emergency department
EMS: emergency medical services
NEDI: National Emergency Department Inventory
RDHRS: Regional Disaster Health Response System
REDCap: Research Electronic Data Capture

## Data Availability

Study data were collected with the promise that individual hospital or emergency department data would not be made publicly available. Thus, the data sets generated during and analyzed during this study have restricted access and are not publicly available. Limited data are available from the corresponding author on reasonable request.
