# Supplementary material for: Hospital-Level Implementation Barriers, Facilitators, and Willingness to Use a New Regional Disaster Teleconsultation System: Cross-Sectional Survey Study
Source: JMIR Public Health Surveill. 2023 Jun 27;9:e44164. doi: 10.2196/44164 (PMC10337359; doi:10.2196/44164)
Supplement: Multimedia Appendix 1 [file publichealth_v9i1e44164_app1.docx]

## **Supplemental Appendix**

# **Hospital-level Implementation Barriers, Facilitators, and Willingness to Use a New Regional Disaster Teleconsultation System: Cross-sectional Survey Study**

**Authors**: Tehnaz P. Boyle, MD, PhD^1^; Krislyn M. Boggs, MPH^2^; Jingya Gao, MS^2^; Maureen McMahon, RN, BSN, MS^3^; Rachel Bedenbaugh, MPH^4^; Lauren Schmidt, MPH^4^; Kori S. Zachrison, MD, MSc^2^; Eric Goralnick, MD, MS^5^; Paul D. Biddinger, MD^2,4^ and Carlos A. Camargo, Jr, MD, DrPH^2^

1–Department of Pediatrics, Boston Medical Center, Boston, MA

2–Department of Emergency Medicine, Massachusetts General Hospital, Boston, MA

3–Department of Emergency Management, Boston Medical Center, Boston, MA

4–Center for Disaster Medicine, Massachusetts General Hospital, Boston, MA

5–Department of Emergency Medicine, Brigham and Women’s Hospital, Boston, MA

## **Contents**

- RDHRS-NEDI 2021 Survey
- Supplement Table 1. Lack of Access to Disaster-Relevant Specialists by Emergency Department Characteristics

RDHRS-NEDI 2021 Survey

*The goal of this study is to collect information from all New England hospitals to guide future implementation of a new federally funded disaster telehealth system to support regional disaster health response across state lines. This system would provide immediate teleconsultation services to connect local healthcare providers with remote clinical specialists via video calls during a disaster.*

1. How does your hospital currently receive notification of a large-scale or state-wide no-notice emergency event? Check ALL that apply:
   1. EMS or public safety system notification (e.g., via EMS radio in the ED)
   2. Hospital network emergency notification system activated at the state level (e.g., by a state department of public health)
   3. Hospital network emergency notification system activated at a regional or county level within a state (e.g., by a regional healthcare coalition)
   4. Media notification (e.g., social media platforms)
   5. Other ________
2. In a large-scale no-notice emergency event, your hospital may receive multiple patients with severe injury or illness requiring specialty care for whom transfer to a specialized facility is delayed. I am now going to read you a list of clinical specialists. Does your hospital have access to the following specialists for consultation in-person, via telephone, or via telehealth within the first 4 hours of such an event? Check ALL that apply:

*(If asked: Specialists include physicians, nurses, pharmacists, or other clinical providers who have advanced training and clinical experience in these areas).*

- 1. Toxicology specialists (e.g., during a mass exposure to hazardous materials)
  2. Radiation or nuclear medicine specialists (e.g., for large-scale radiation/nuclear emergencies)
  3. Trauma specialists (e.g., for a large-scale mass casualty event)
  4. Burn specialists (e.g., for a large-scale burn/blast event)
  5. Infectious disease specialists (e.g., for a bio-terrorist attack or high consequence infectious disease)
  6. Critical care specialists (e.g., for a high-acuity mass illness event such as COVID-19)

1. *The disaster teleconsultation system enables peer-to-peer connections between local clinicians and remote specialists via video calls. These specialists are available to advise frontline clinicians on highly specialized clinical care in the first hours after a no-notice emergency event. All treatment decisions would remain in the purview of the frontline practitioner.*

Considering this definition of the disaster teleconsultation system, would your hospital require any disaster or emergency credentialing and privileging of the remote specialists before system use?

🞏 No (**If No, skip to Q4**)

🞏 Yes (**If Yes, proceed to Q3A**):

3A. Does your hospital have disaster/emergency credentialing and privileging procedures written in the medical staff bylaws?

🞏 No (**If No, skip to Q3E**)

🞏 Yes (**If Yes, proceed to Q3B-Q3D**)

3B. Which disaster/emergency credentialing and privileging process does your hospital currently use? Check ALL that apply:

🞏 Credentialing-by-proxy (*If asked: This process allows a hospital receiving telehealth services to accept the proof of credentials and privileging decisions made by the specialist’s host institution providing essential requirements are met.*)

🞏 Disaster privileges (*If asked: Disaster privileges are temporary privileges a hospital may grant after activating its emergency operations plan (EOP) in response to a disaster event where additional staff are needed to meet immediate patient needs*.)

🞏 Other process _________________________________________________________

3C. What is the expected timeline to complete your disaster/emergency privileging procedure as written in your medical staff bylaws?

1. Less than 4 hours
2. 4-24 hours
3. 25-72 hours
4. Greater than 72 hours

3D. To your knowledge, has your hospital utilized the disaster/emergency privileging process during a major event in the last 20 years?

🞏 No
🞏 Yes*

*If yes to Q2D, was your hospital successful in completing disaster/emergency privileging in less than 4 hours?

🞏 No
🞏 Yes

3E. During a disaster, as part of your hospital’s existing disaster privileging processes, would your hospital be willing to privilege providers with pre-verified credentials if made available by a third-party system such as Provider Bridge?

*(If asked: Provider Bridge is a secure online platform developed by the Federation of State Medical Boards and HRSA to streamline mobilization of healthcare professionals during public health emergencies. Providers can upload their credentials and be quickly pre-vetted for hospitals seeking volunteer health professionals during a disaster.)*

🞏 No

🞏 Yes

1. Does your hospital have reliable internet or cellular service to support video streaming in clinical spaces?

🞏 No

🞏 Yes (**If Yes, proceed to Q4A**)

4A. Can your hospital maintain reliable cellular service in clinical spaces if the hospital internet connection is disrupted?

🞏 No

🞏 Yes

1. In the event of a disaster, does your hospital have a contingency plan for loss of internet or cellular service (e.g., FirstNet subscription or WPS cards)?

🞏 No

🞏 Yes

1. If the disaster teleconsultation system was activated to support a large-scale or state-wide no-notice emergency event affecting your hospital, how likely would it be that your hospital would use this system to access specialists?

🞏 Very likely

🞏 Somewhat likely

🞏 Somewhat unlikely*

🞏 Very unlikely*

***If somewhat unlikely/very unlikely, proceed to Q6A**

6A. In addition to the barriers already discussed, which of the following reasons make it unlikely for your hospital to use the disaster teleconsultation system? Check ALL that apply:

🞏 Concern about time delays in care related to system use

🞏 Reluctance to use new or unfamiliar technology or systems during a disaster

🞏 Hospital information system security restrictions (e.g., firewalls or restricted URLs) would prevent clinicians from accessing the service

🞏 HIPAA or privacy concerns about sharing patient information

🞏 Medicolegal liability concerns with system usage

🞏 Other*

*If Other, please explain__________________

*Thank you for participating in this study.*

Table S1. Lack of access to disaster-relevant specialists by emergency department characteristics

| No access to in-person, telephone, or telehealth consultation within 4-hours | Toxicology specialists | Radiation or nuclear medicine specialists | Trauma specialists | Burn specialists | High-consequence infectious disease specialists | Critical care specialists | No access to any specialist |
| --- | --- | --- | --- | --- | --- | --- | --- |
|  | n (%) | n (%) | n (%) | n (%) | n (%) | n (%) | n (%) |
| Overall population (n=164) | 30 (18) | 25 (15) | 20 (12) | 40 (24) | 7 (4) | 6 (4) | 2 (1) |
| Annual ED visit volume |  |  |  |  |  |  |  |
| < 10,000 (n=22) | 5 (23) | 1 (5) | 1 (5) | 5 (23) | 2 (9) | 1 (5) | 0 (0) |
| 10,000-19,999 (n=46) | 10 (22) | 13 (28) | 10 (22) | 11 (24) | 3 (7) | 4 (9) | 1 (2) |
| 20,000-39,999 (n=48) | 11(23) | 7 (15) | 8 (17) | 17 (35) | 2 (4) | 1 (2) | 1 (2) |
| ≥40,000 (n=48) | 4 (8) | 4 (8) | 1 (2) | 7 (15) | 0 (0) | 0 (0) | 0 (0) |
| Critical access hospital |  |  |  |  |  |  |  |
| Yes (n=32) | 8 (25) | 6 (19) | 3 (9) | 7 (22) | 2 (6) | 1(3) | 0 (0) |
| No (n=132) | 22 (17) | 19 (14) | 17 (13) | 33 (25) | 5 (4) | 5 (4) | 2 (2) |
| Urban status |  |  |  |  |  |  |  |
| Urban (n=142) | 29 (20) | 21 (15) | 18 (13) | 37 (26) | 5 (4) | 4 (3) | 2 (1) |
| Rural (n=22) | 1(5) | 4 (18) | 2 (9) | 3 (14) | 2 (9) | 2 (9) | 0 (0) |

Notes: Three participants did not answer the “access to disaster-relevant specialists” question.
